# Advances in Gynecological Diseases

**DOI:** 10.3390/jcm14196988

**Published:** 2025-10-02

**Authors:** Guglielmo Stabile, Laura Vona, Stefano Restaino, Martina Arcieri, Luigi Nappi

**Affiliations:** 1 Department of Medical and Surgical Sciences, Institute of Obstetrics and Gynecology, University of Foggia, 71121 Foggia, Italy; lauravona@hotmail.it (L.V.); luigi.nappi@unifg.it (L.N.); 2 Clinic of Obstetrics and Gynecology, “Santa Maria della Misericordia” University Hospital, Azienda Sanitaria Universitaria Friuli Centrale, 33100 Udine, Italy; restaino.stefano@gmail.com (S.R.); martina.arcieri@asufc.sanita.fvg.it (M.A.)

In recent decades, gynecology has undergone a profound transformation driven by technological, pharmacological, and biotechnological innovations that have fundamentally changed the diagnosis, management, and treatment of numerous gynecologic conditions. These advancements have facilitated increasingly precise, safe, and personalized care, improving outcomes for women across the spectrum of reproductive and pelvic health. Among the areas that have benefited most are the management of uterine fibroids, endometriosis, fertility disorders, pelvic organ prolapse, and complex gynecologic malignancies [1]. This progress has been enabled by the integration of advanced surgical techniques such as operative hysteroscopy, minilaparoscopy, Vaginal Natural Orifice Transluminal Endoscopic Surgery (vNOTES), and intraoperative ultrasound, alongside novel pharmacological therapies, including gonadotropin-releasing hormone (GnRH) antagonists, and emerging regenerative medicine strategies, including the use of platelet-rich plasma (PRP).

Uterine fibroids, also known as leiomyomas, are the most common benign tumors in women of reproductive age. These tumors may present with a spectrum of clinical manifestations, including abnormal uterine bleeding, pelvic pain, and infertility [2]. Submucosal fibroids, in particular, protrude into the endometrial cavity and are closely associated with impaired implantation and reduced fertility. The advent of operative hysteroscopy has revolutionized the management of submucosal fibroids by providing direct access to the uterine cavity via the cervical canal without the need for abdominal incisions [3]. This technique allows for the precise resection of fibroids using bipolar resectoscopes or mechanical morcellation systems, preserving the uterine architecture, minimizing surgical complications, and facilitating rapid postoperative recovery while maintaining reproductive potential. The ability to perform these procedures in an outpatient or office-based setting further enhances patient comfort and reduces healthcare costs [4]. Evidence from multicenter studies and consensus guidelines, including those from the European Society for Gynaecological Endoscopy (ESGE), consistently supports operative hysteroscopy as the gold standard for the management of symptomatic submucosal fibroids, particularly in women desiring future fertility [5]. The limitations of hysteroscopic management include technical challenges in large or sessile fibroids and the need for operator expertise; however, ongoing technological improvements, including miniaturized instruments and enhanced visualization systems, continue to expand its applicability.

In parallel with surgical innovation, pharmacologic therapy has evolved significantly, particularly with the development of next-generation GnRH antagonists such as relugolix, elagolix, and linzagolix. These agents act rapidly and reversibly to suppress gonadotropin secretion, thereby reducing estrogen and progesterone levels, which in turn leads to a decrease in fibroid volume and alleviation of abnormal uterine bleeding [6]. Compared with traditional GnRH analogs, these antagonists offer improved tolerability and the possibility of add-back therapy to mitigate hypoestrogenic side effects, including bone mineral density loss and vasomotor symptoms. Clinical trials, such as the LIBERTY study, have demonstrated the efficacy of these agents in reducing fibroid size and controlling menorrhagia, while maintaining patient safety [7]. Beyond fibroids, GnRH antagonists have shown substantial efficacy in managing endometriosis, a condition characterized by ectopic endometrial tissue causing chronic pelvic pain, dysmenorrhea, dyspareunia, and infertility. The suppression of ovarian hormone production with these agents effectively reduces the proliferation of ectopic endometrial lesions and alleviates associated pain, thereby improving quality of life [8]. Importantly, the use of medical therapy in endometriosis reduces the need for repeated surgical interventions, which have been demonstrated to negatively affect ovarian reserve and compromise pelvic tissue integrity, thereby preserving reproductive potential and limiting long-term morbidity.

Complementary to pharmacologic therapy, intraoperative imaging, particularly ultrasound, has emerged as a valuable adjunct in the surgical management of endometriosis. Intraoperative ultrasound enables the precise localization of deep infiltrating endometriotic lesions affecting the bladder, bowel, uterosacral ligaments, or other pelvic structures [9]. This image-guided approach enhances surgical accuracy, facilitates complete lesion excision, and reduces the risk of recurrence, particularly in complex cases where anatomical distortion is significant [10]. By combining medical therapy with intraoperative imaging, clinicians can adopt an integrated, multidisciplinary approach that optimizes both conservative and surgical management, improving outcomes while minimizing the invasiveness of interventions.

Another frontier in gynecologic care is regenerative medicine, with platelet-rich plasma (PRP) representing one of the most promising applications. PRP is an autologous concentration of platelets enriched with growth factors, including platelet-derived growth factor (PDGF), transforming growth factor-beta (TGF-β), vascular endothelial growth factor (VEGF), and epidermal growth factor (EGF), which collectively stimulate tissue repair, angiogenesis, and cellular proliferation [11]. In reproductive medicine, PRP has been employed to enhance endometrial thickness in women with refractory thin endometrium, improving implantation rates and pregnancy outcomes in assisted reproductive technology cycles [12]. Additionally, intraovarian PRP administration is under investigation as a strategy to stimulate follicular development in women with diminished ovarian reserve or premature ovarian insufficiency [13]. PRP also offers potential therapeutic benefits in pelvic organ prolapse by promoting tissue regeneration and improving the trophism of vaginal and perineal tissues, particularly when used alongside pelvic floor rehabilitation or local estrogen therapy. Early clinical studies suggest that PRP may reduce the need for surgical intervention in selected cases, though larger randomized trials are required to establish standardized protocols and long-term efficacy [14].

Minimally invasive surgery has also experienced significant advancement with the development of minilaparoscopy. Utilizing instruments as small as 2–3 mm in diameter, minilaparoscopy permits complex procedures with reduced surgical trauma, less postoperative pain, and superior cosmetic outcomes [15]. In the context of gynecologic oncology, minilaparoscopy has been applied to the management of ovarian cancer, enabling accurate tumor staging, the assessment of resectability in advanced disease, and, in specialized centers, interval or primary debulking procedures [16]. By minimizing tissue manipulation and preserving the abdominal wall’s integrity, minilaparoscopy reduces hospitalization time and postoperative complications, while providing comparable oncologic outcomes to conventional laparoscopy or laparotomy in selected patient populations. Its applicability is not limited to oncology; minilaparoscopy has also been utilized successfully in the management of benign gynecologic conditions, including deep infiltrating endometriosis, complex adhesiolysis, and hysterectomy for benign indications, demonstrating versatility across a wide range of surgical scenarios [17].

In addition to minilaparoscopy, Vaginal Natural Orifice Transluminal Endoscopic Surgery (vNOTES) has emerged as a novel approach in minimally invasive gynecologic surgery, particularly advantageous in obese patients. vNOTES utilizes the vaginal canal to access the peritoneal cavity, eliminating abdominal incisions and reducing postoperative morbidity [18]. Recent systematic reviews and clinical studies have demonstrated the feasibility and safety of vNOTES in obese populations, as well as for large uterus, leading to reduced operative times, lower postoperative pain, shorter hospital stays, and fewer complications compared to conventional laparoscopy. In a multicenter study involving obese patients with a body mass index exceeding 30 kg/m^2^, all vNOTES procedures were successfully completed without conversion to laparotomy, with minimal incidence of bladder or rectal injuries [19]. Despite the technical challenges posed by increased pelvic adiposity, careful patient selection, preoperative planning, and specialized surgical expertise enable the safe and effective implementation of this approach. By offering superior cosmetic outcomes, reduced postoperative discomfort, and enhanced recovery, vNOTES complements minilaparoscopy and represents a significant step forward in minimally invasive gynecologic surgery, particularly in high-risk populations such as obese patients.

A particularly challenging area in gynecology is the management of intrauterine ectopic pregnancies, including scar pregnancies and other abnormal implantation sites within the uterus, such as cervical or intramural locations. These pregnancies, while rare, carry a high risk of hemorrhage and significant morbidity if not managed appropriately [20]. Recent clinical experience has demonstrated that conservative approaches, combining pharmacologic therapy and operative hysteroscopy, can effectively manage these conditions while preserving fertility. Scar pregnancies, occurring within a prior cesarean section scar, have been successfully treated with a combination of methotrexate and mifepristone, which promotes regression of the gestational sac and reduces the need for invasive surgical interventions [21]. Conversely, operative hysteroscopy allows the direct removal of gestational tissue under visual guidance, with effective hemostasis and the option to integrate adjunctive measures such as local tamponade or uterine artery embolization in complex cases [22]. This combined pharmacologic and hysteroscopic approach represents a paradigm shift in the management of intrauterine ectopic pregnancies, emphasizing conservative, fertility-preserving strategies that minimize surgical morbidity.

The integration of these therapeutic modalities—advanced minimally invasive surgery, targeted pharmacologic therapy, regenerative medicine, and image-guided surgical techniques—reflects a broader trend toward personalized gynecologic care. Personalized approaches enable clinicians to tailor interventions based on patient-specific factors, including age, reproductive goals, comorbidities, and the anatomical complexity of disease.

In conclusion, the past decades have witnessed unprecedented advances in gynecology, characterized by the convergence of minimally invasive surgical techniques, targeted pharmacological interventions, regenerative medicine, and precision imaging. Operative hysteroscopy has become the standard for submucosal fibroid management, next-generation GnRH antagonists provide effective and tolerable therapy for fibroids and endometriosis, PRP offers regenerative potential for fertility and prolapse, minilaparoscopy and vNOTES enable complex oncologic and benign interventions with reduced morbidity, and intraoperative ultrasound enhances the precision of endometriosis surgery. Conservative management strategies for intrauterine ectopic pregnancies, combining pharmacologic therapy and hysteroscopy, exemplify the emphasis on fertility preservation and minimally invasive care. Collectively, these advancements illustrate a paradigm shift toward personalized, integrative gynecologic care, with ongoing innovations in regenerative medicine and imaging technologies poised to further improve outcomes. The continued evolution of these approaches promises tangible, long-term benefits for women’s reproductive health, quality of life, and overall well-being, establishing gynecology as one of the most dynamic and innovative fields in modern medicine.
